# Supplementary material for: Defining a Water-Soluble Formulation of Arachidonic Acid as a Novel Ferroptosis Inducer in Cancer Cells
Source: Biomolecules. 2024 May 4;14(5):555. doi: 10.3390/biom14050555 (PMC11118058; doi:10.3390/biom14050555)
Supplement: Supplementary file 1 [file biomolecules-14-00555-s001.zip › biomolecules-2925472-supplementary figures.pdf]

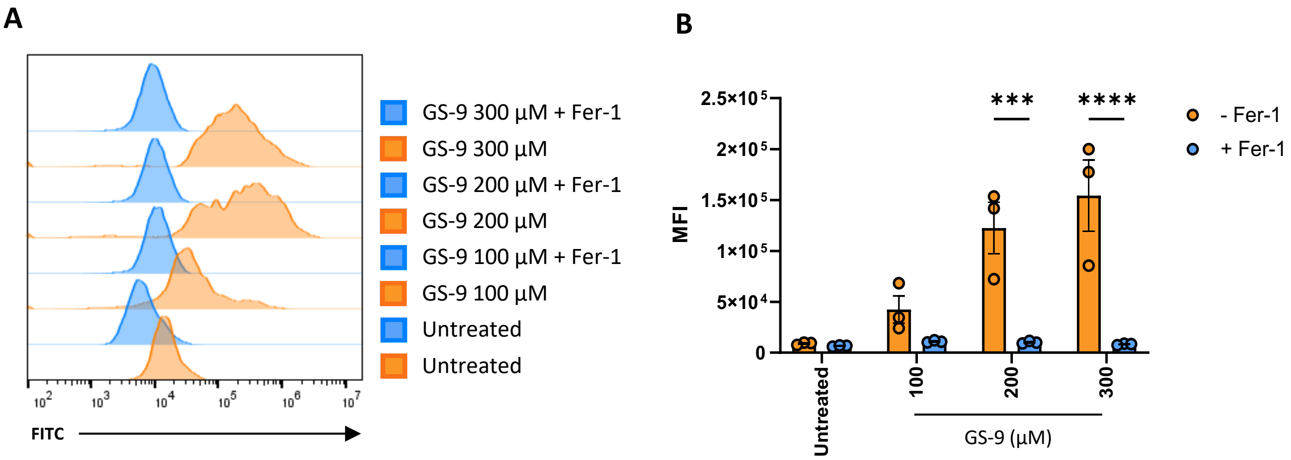

Supplementary Figure S1. GS-9 induces lipid peroxidation in Jurkat Cells. (A) Jurkat cells were stained with C11-BODIPY and treated with increasing concentrations of GS-9 for 24 h. Oxidised BODIPY C11 (FITC) levels were plotted as histograms after flow cytometry analysis. C11-BODIPY MFI was quantified in **(B)** (n=3 ± S.E.M., 2way Anova, Šídák's Multiple Comparisons, *** $p = 0.0006$, **** $p < 0.0001$).

A

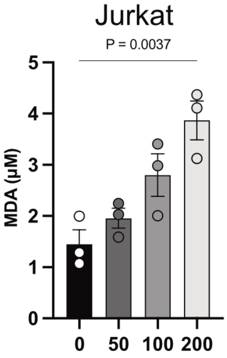

Supplementary Figure S2. GS-9 induces MDA Accumulation in Jurkat Cells. (A) Jurkat cells were treated with increasing concentrations of GS-9 for 24 h before MDA levels were analysed (n = 3, ± S.E.M., one-way ANOVA, Tukey's multiple comparison test).

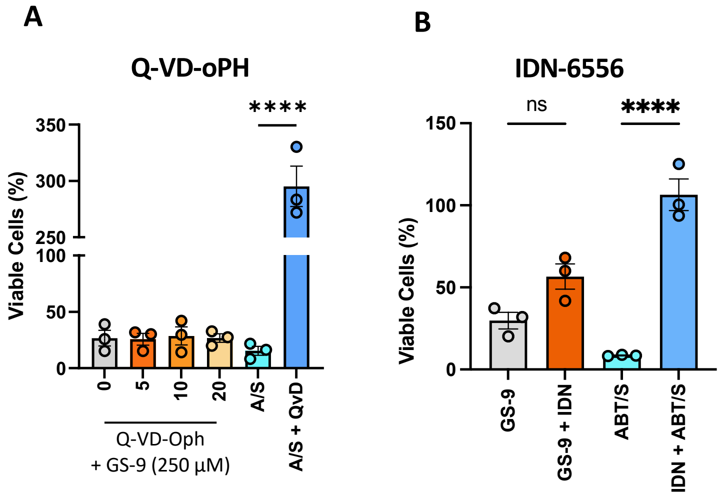

Supplementary Figure S3. Pan-caspase inhibitors do not protect from GS-9 killing in Jurkat cells. Jurkat cells were treated with increasing concentrations of **(A)** Q-VD-oPH or **(B)** IDN-6556 at 5 µM in the presence of 250 µM GS-9 as well as a BH3 mimetic apoptosis positive control before undergoing MTS assays to determine viability as a percentage of vehicle controls (Q-VD-oPH: n = 3, mean ± S.E.M., One-way Anova, Tukey's Multiple Comparison, $p > 0.0001$) (IDN-6556: n = 3, mean ± S.E.M., One-way Anova, Tukey's Multiple Comparison, $p > 0.0001$).

A

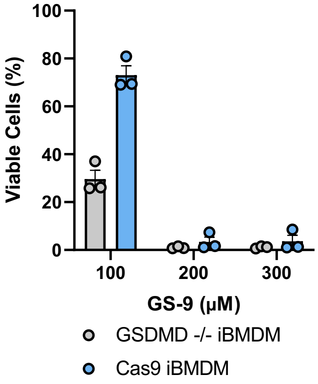

Supplementary Figure S4. GS-9 does not induce Pyroptosis. (A) iBMDM Cas9 and GSDMD$^{/-}$ cells were treated with increasing concentrations of GS-9 before undergoing an MTT assay to determine viability (n = 3, ±S.E.M).

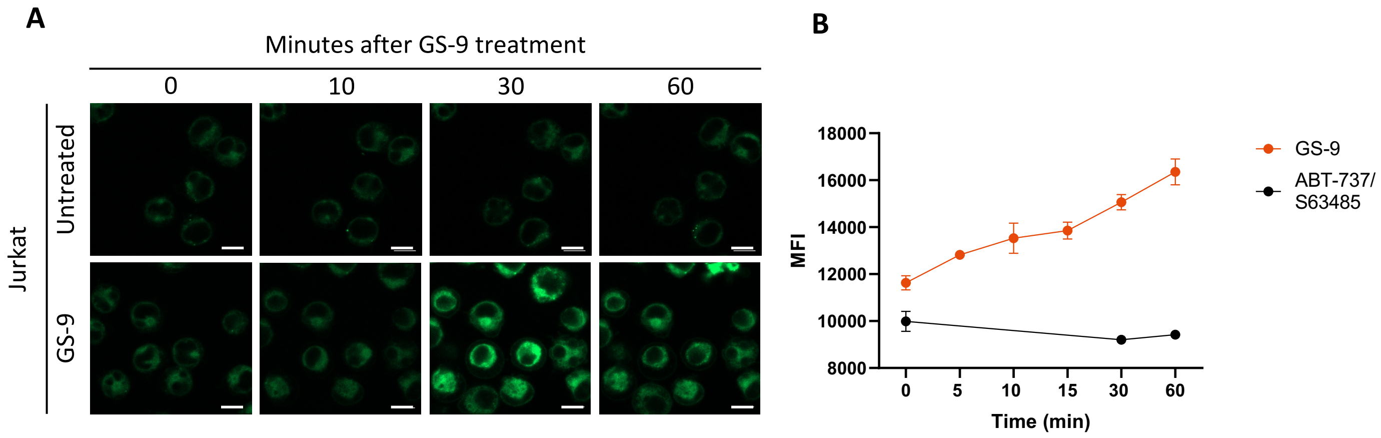

Supplementary Figure S5. GS-9 localises intracellularly at lipid droplets in Jurkat cells. (A) Confocal time course microscopy analysis of Jurkat cells stained with BODIPY and treated with GS-9 (scale bar = 10 µm; images representative of 3 experiments). **(B)** Jurkat cells were stained with BODIPY and treated with GS-9 prior to analysis by flow cytometry to determine lipid droplet levels as indicated by BODIPY MFI (n = 3, ±S.E.M.).

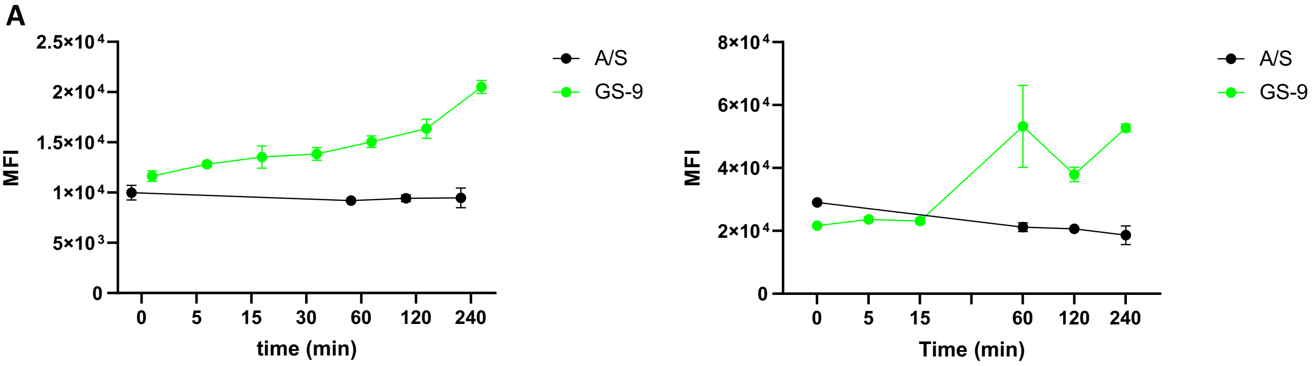

Supplementary Figure S6. GS-9 Uptake Replicates. (A) Additional replicates showing GS-9 uptake, A549 cells were stained with BODIPY and treated with GS-9 prior to analysis by flow cytometry to determine lipid droplet levels as indicated by BODIPY MFI (n = 3, ±S.E.M).

A

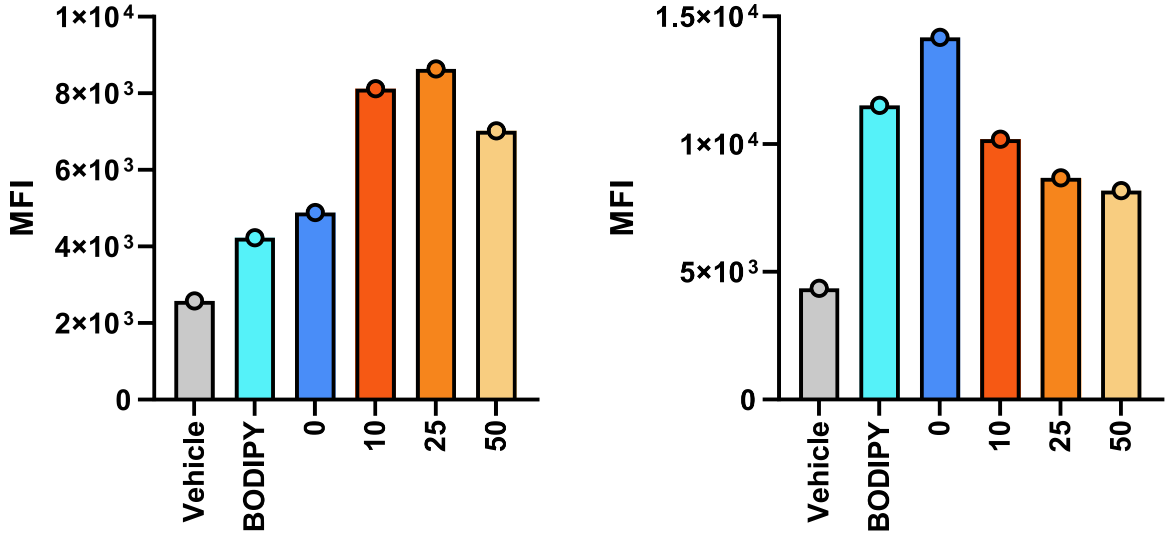

Supplementary Figure S7. Effect of Grassofermata on GS-9 Uptake (A) A549 cells were stained with BODIPY and treated with GS-9 and increasing concentrations of Grassofermata (GF) for 4 h. BODIPY levels were quantified as MFI (n = 3, ±S.E.M).
