# Supplementary figures and images for: Defining a Water-Soluble Formulation of Arachidonic Acid as a Novel Ferroptosis Inducer in Cancer Cells
Source: Biomolecules. 2024 May 4;14(5):555. doi: 10.3390/biom14050555 (PMC11118058; doi:10.3390/biom14050555)

Figure 4 A Original Western Blots

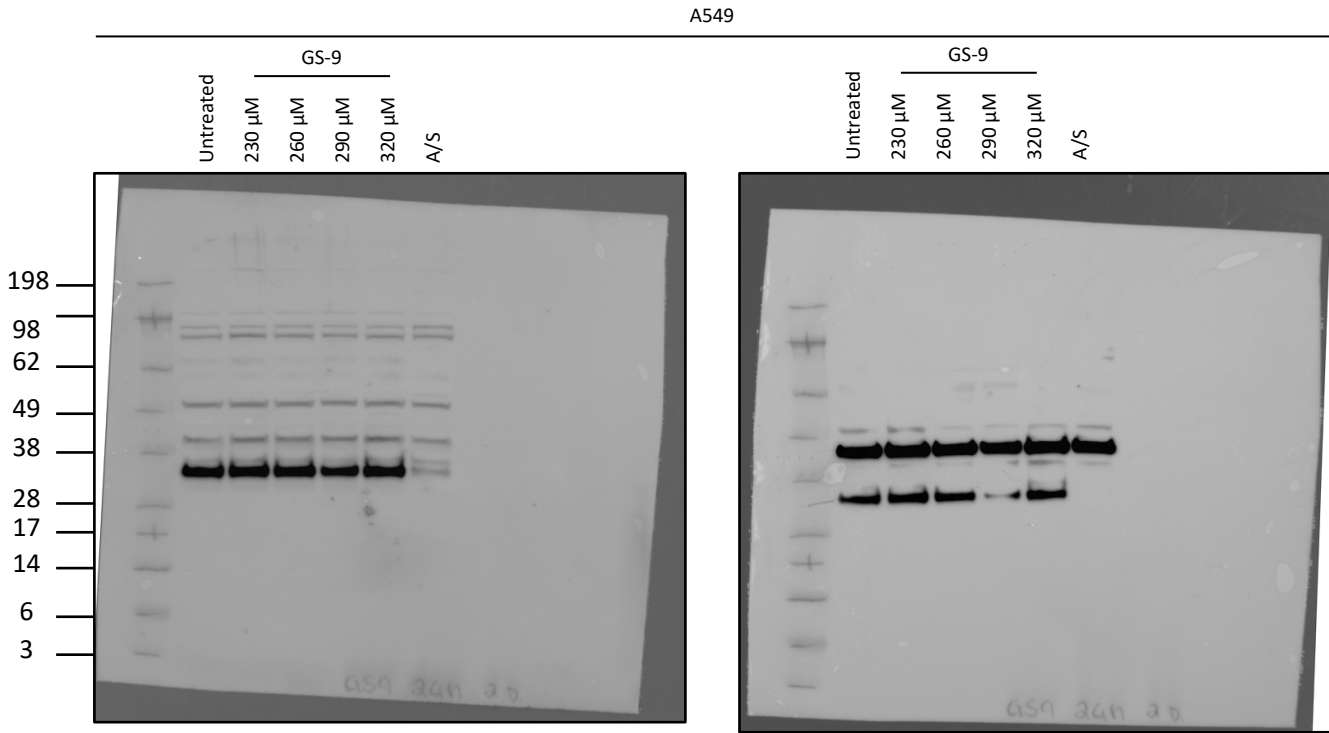

Figure 5 B Original Western Blots

HT-29 (6h)

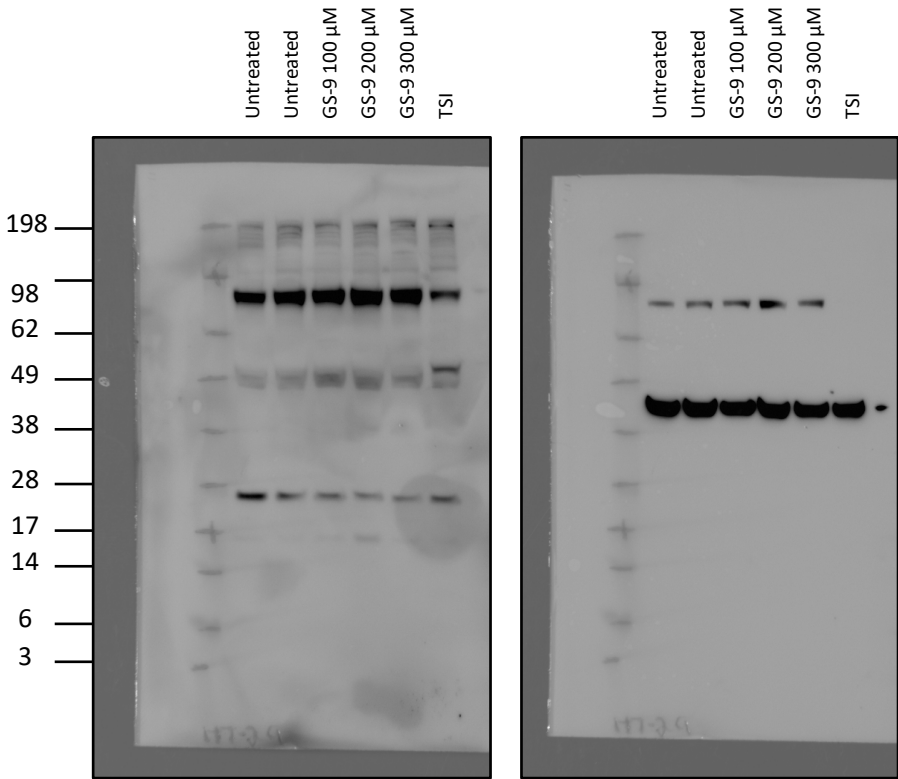

Supplement: Supplementary file 1 [file biomolecules-14-00555-s001.zip › biomolecules-2925472-supplementary file S1.pdf]
